# Challenges in Malaria Diagnosis and Treatment in Kinshasa Province, Democratic Republic of the Congo

**DOI:** 10.1093/cid/ciag262

**Published:** 2026-04-18

**Authors:** Ruthly François-Zafka, Daniel Westreich, Kristin Banek, Melchior Mwandagalirwa Kashamuka, Sam J White, Joseph A Bala, Marthe Nkalani, Georges Kihuma, Joseph Atibu, Georges E Mahilu, Kyaw L Thwai, Jack Kokolomami, Michael Emch, Jessie Edwards, Jonathan J Juliano, Antoinette Tshefu, Jonathan B Parr

**Affiliations:** Department of Epidemiology, Gillings School of Global Public Health, University of North Carolina at Chapel Hill, Chapel Hill, North Carolina, USA; Department of Epidemiology, Gillings School of Global Public Health, University of North Carolina at Chapel Hill, Chapel Hill, North Carolina, USA; Department of Epidemiology, Celia Scott Weatherhead School of Public Health and Tropical Medicine, Tulane University, New Orleans, Louisiana, USA; Department of Epidemiology and Biostatistics, Kinshasa School of Public Health, University of Kinshasa, Kinshasa, Democratic Republic of the Congo; Institute for Global Health and Infectious Diseases, University of North Carolina at Chapel Hill, Chapel Hill, North Carolina, USA; Department of Epidemiology and Biostatistics, Kinshasa School of Public Health, University of Kinshasa, Kinshasa, Democratic Republic of the Congo; Department of Epidemiology and Biostatistics, Kinshasa School of Public Health, University of Kinshasa, Kinshasa, Democratic Republic of the Congo; Department of Epidemiology and Biostatistics, Kinshasa School of Public Health, University of Kinshasa, Kinshasa, Democratic Republic of the Congo; Department of Epidemiology and Biostatistics, Kinshasa School of Public Health, University of Kinshasa, Kinshasa, Democratic Republic of the Congo; Department of Epidemiology and Biostatistics, Kinshasa School of Public Health, University of Kinshasa, Kinshasa, Democratic Republic of the Congo; Institute for Global Health and Infectious Diseases, University of North Carolina at Chapel Hill, Chapel Hill, North Carolina, USA; Department of Epidemiology and Biostatistics, Kinshasa School of Public Health, University of Kinshasa, Kinshasa, Democratic Republic of the Congo; Department of Epidemiology, Gillings School of Global Public Health, University of North Carolina at Chapel Hill, Chapel Hill, North Carolina, USA; Department of Geography, University of North Carolina at Chapel Hill, Chapel Hill, North Carolina, USA; Department of Epidemiology, Gillings School of Global Public Health, University of North Carolina at Chapel Hill, Chapel Hill, North Carolina, USA; Department of Epidemiology, Gillings School of Global Public Health, University of North Carolina at Chapel Hill, Chapel Hill, North Carolina, USA; Institute for Global Health and Infectious Diseases, University of North Carolina at Chapel Hill, Chapel Hill, North Carolina, USA; Department of Epidemiology and Biostatistics, Kinshasa School of Public Health, University of Kinshasa, Kinshasa, Democratic Republic of the Congo; Institute for Global Health and Infectious Diseases, University of North Carolina at Chapel Hill, Chapel Hill, North Carolina, USA

**Keywords:** malaria, *Plasmodium falciparum*, diagnosis, treatment, DRC

## Abstract

**Background:**

Effective malaria case management relies on accurate diagnosis and appropriate treatment. However, rapid diagnostic test (RDT) performance varies, and presumptive treatment remains common. We quantified malaria misdiagnosis and inappropriate treatment and assessed adherence to treatment guidelines in a high-burden setting.

**Methods:**

We leveraged samples collected during a cohort study of children and adults from 2015 to 2022 across 7 sites in Kinshasa Province, Democratic Republic of the Congo (DRC). We estimated the cumulative incidence and prevalence of false-positive and false-negative RDT results using polymerase chain reaction (PCR) as the gold standard and identified correlates of misdiagnosis and inappropriate treatment using Kaplan–Meier and generalized estimating equations.

**Results:**

Among 2269 participants, the 1-year cumulative incidences of false-positive and false-negative RDTs were 23.1% and 17.8%, respectively; by 7 years, these rose to 77.7% and 83.4%. Misdiagnosis (false-positive or false-negative RDT) was associated with age, recent treatment, transmission intensity, and parasite density—factors that could inform refinements to treatment guidelines. About 58% of RDT-negative participants received antimalarial treatment at a clinic visit, while 4% of RDT-positive participants did not. Fever significantly reduced adherence to test-and-treat guidelines.

**Conclusions:**

Most participants experienced malaria misdiagnosis by RDT over the course of the study, underscoring the limitations of currently used RDTs and opportunities for improved clinical decision-making. While RDTs remain essential for malaria case management, clinicians should consider contextual factors when interpreting results. Efforts to improve adherence to guidelines, which require a positive diagnostic test result before treatment, and management of non-malarial febrile illness are needed in the DRC and similar high-burden settings.

## Teaser

We investigated challenges to effective malaria case management in a 2015–2022 cohort of 2269 participants in Kinshasa Province, DRC. Nearly all participants were misdiagnosed at least once by rapid diagnostic tests, and presumptive treatment was common among febrile, RDT-negative participants.

## BACKGROUND

Malaria is a preventable and treatable disease, yet it remains a major public health issue in low- and middle-income countries. In 2023, sub-Saharan Africa (SSA) accounted for 94% and 95% of the world's 263 million malaria cases and 597 000 deaths, respectively [1]. Effective case management is crucial for reducing malaria's impact and relies on prompt and accurate diagnosis and treatment [2]. In SSA, most efforts focus on identifying and treating people infected with *Plasmodium falciparum*, the most common and deadliest malaria-causing parasite species.

Falciparum malaria is primarily diagnosed using rapid diagnostic tests (RDTs), most of which detect *P. falciparum* histidine-rich protein 2 (HRP2) antigen, alone or with pan-*Plasmodium* lactate dehydrogenase [1, 3–5]. Compared to microscopy, considered the gold standard, RDTs require less technical expertise and infrastructure [4, 5]. Despite the ease of use, high sensitivity, and cost-effectiveness, RDTs are imperfect tools [4, 5]. Rapid diagnostic test performance is affected by operator error, *hrp2* gene deletion, and endemicity which influences parasite density and the likelihood of persistent HRP2 antigenemia after treatment [5–9]. These factors can result in misdiagnosis and improper care.

The World Health Organization recommends parasitological confirmation of all suspected malaria cases prior to treatment [2]. This test-and-treat policy was informed by the decreasing proportion of malaria-attributable febrile illnesses and the need to mitigate the emergence of antimalarial drug resistance [10]. Large-scale RDT deployment reduced drug prescriptions in some endemic countries [4, 11]. However, treatment based on clinical diagnosis is still common, with various countries reporting more than half of RDT-negative patients receiving antimalarial drugs, particularly symptomatic children in high-transmission settings [12–14]. Besides unnecessary drug side-effect exposure, overtreatment without making a definitive diagnosis can have disastrous consequences, with higher mortality observed among RDT-negative children who received antimalarial treatment [12].

These issues are important in the Democratic Republic of the Congo (DRC), where malaria remains a leading cause of morbidity and mortality [15]. Studies have identified false-negative RDTs due to *hrp2* deletion in some provinces and explored impacts of RDT performance on case management [16–22]. However, the association between misdiagnosis and inappropriate treatment in this high-burden country with substantial spatial heterogeneity in malaria prevalence is unclear. We leveraged a 7-year longitudinal cohort of children and adults across 7 sites of varying transmission intensity to estimate the extent of malaria misdiagnosis, identify correlates of misdiagnosis, and investigate adherence to test-and-treat guidelines.

## METHODS

### Study Design and Population

**Figure 1.**
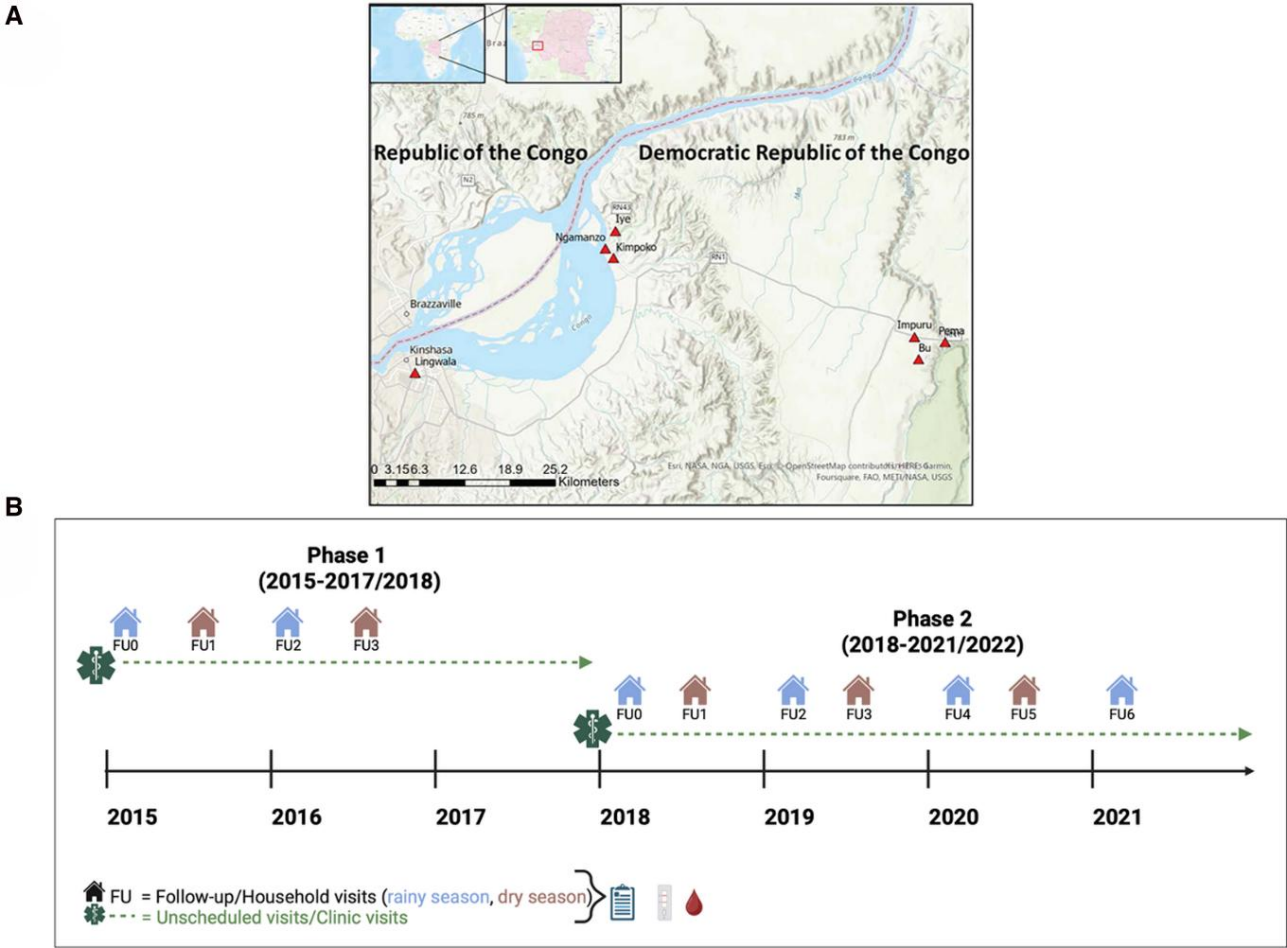
Study sites, design, and retention for the Kinshasa malaria longitudinal cohort study of 2269 children and adults between 2015 and 2022. *A*, Seven study sites were selected to reflect different malaria transmission intensity in Kinshasa Province: Lingwala (urban, low transmission); Ngamanzo, Iye, Kimpoko (peri-urban, moderate transmission); and Bu, Impuru, Pema (rural, high transmission) [20]. *B*, Households were visited twice yearly, alternating between the rainy and dry seasons (blue and brown colors, respectively). During Phase 1, baseline and 3 follow-up household visits were conducted (FU0 to FU3); during Phase 2, baseline and 6 follow-up visits were conducted (FU0 to FU6). During these visits and at unscheduled clinic visits for malaria symptoms, a questionnaire on demographics and malaria-related factors was administered, along with blood collection for malaria rapid diagnostic tests and dried blood spots.

This study leverages 2 cohort studies conducted at 7 sites across 3 health areas of varying malaria prevalence in Kinshasa Province, DRC (Figure 1*A*) [20]. The study design and population for the DRC malaria longitudinal study Phase 1 (2015–2018) and Phase 2 (2018–2022) have been previously described [7, 23–25]. Briefly, children and adults from representative households at each site were invited to participate. Individuals who gave informed consent or assent were surveyed twice yearly, once during the rainy season (September–May) and once during the dry season (June–August) (active surveillance). Questionnaires were administered to collect data on demographics, household characteristics, bednet use, and malaria diagnosis, symptoms, and treatment. Malaria RDTs were performed, and dried blood spots (DBS) were collected. The RDT-positive participants were referred for treatment at local health facilities. Similar data and samples were collected from participants experiencing malaria-like symptoms who presented to health facilities at the study sites (passive surveillance). At the end of Phase 1, participants were invited to participate in Phase 2 and re-enrolled upon consent; new households and participants were also recruited (Figure 1*B*; Supplementary Materials A1).

### *Plasmodium* Detection

*Plasmodium* infection was detected using an SD Bioline Ag P.f./Pan ([05FK60], Alere, Gyeonggi-do, Republic of Korea), then later in the study period, CareStart Malaria Pf HRP2 Ag ([02571], Access Bio, Somerset, NJ) RDT owing to a change in RDT procurement by the national malaria control program in 2019. DNA was extracted from DBS, and real-time quantitative polymerase chain reaction (qPCR) targeting *P. falciparum lactate dehydrogenase* was performed to detect and quantify *P. falciparum* density, with positive calls made as previously described [23, 26]. Quantitative PCR was considered the gold standard for *P. falciparum* infection (detection limit: 5–10 parasites/μL), and qPCR-RDT agreement was calculated using Cohen's Kappa statistic.

### Data Analysis

The primary outcomes for *P. falciparum* infection misdiagnosis were false-positive (qPCR-negative/RDT-positive) and false-negative (qPCR-positive/RDT-negative) RDT results. Cumulative incidences of the first *P. falciparum* infection and first instance of false-positive and false-negative RDT were estimated using Kaplan–Meier curves. We conducted survival analysis using time since study start as the timescale and incorporated stabilized inverse probability of censoring weights to correct for differential loss to follow-up as described in the Supplementary Materials A2. Although death is a competing event for the outcomes, we treated death as a censored event considering the few deaths reported (n = 28 deaths among 2269 participants—4 during Phase 1 and 24 during Phase 2).

We also estimated the proportion of false-positive and false-negative RDTs among all the RDTs performed over the 7-year period and at each household follow-up and clinic visit period. Clinic visit periods covered the start of a household follow-up and ended before the start of the next household follow-up. We identified demographic, clinical, community, and parasite factors associated with false-positive and false-negative RDTs. Bivariate generalized estimating equation (GEE) models with an exchangeable correlation structure were used to estimate crude proportion differences of false-positive and false-negative RDTs across each factor while accounting for repeated measurements within participants and assuming constant correlation between any pair of observations for a participant. A detailed description of these factors is provided in the Supplementary Materials A3. Similar analyses were conducted to identify correlates of RDT diagnosis accuracy by comparing the proportion differences of false-positive versus true-negative RDTs and false-negative versus true-positive RDTs.

To investigate inappropriate treatment, we restricted analyses to the available clinic visit data that included recorded antimalarial drug prescription after an RDT result. We considered both policy and research perspectives. From the policy perspective, which reflects adherence to treatment guidelines, we defined overtreatment as treatment provided to RDT-negative participants or those without an RDT test performed, and undertreatment as treatment withheld from RDT-positive participants. Separate bivariate GEE analyses as described above were conducted to identify correlates of over- and undertreatment.

Given numerous reports of presumptive treatment, we investigated the role of RDT results in treatment prescription and how the presence of fever influences this decision. Specifically, we estimated the probability of treatment among RDT-positive participants compared to RDT-negative participants using GEE with an interaction term between RDT result and fever. Potential confounders were identified using a directed acyclic graph and addressed using inverse probability of treatment weighting (Supplementary Figure 1). For each visit, the treatment and censoring weights were multiplied to generate a combined weight. To estimate appropriate standard errors, 1000 bootstraps were performed.

Descriptive statistics and Sankey plots were used to estimate and visualize the proportion of qPCR-confirmed *P. falciparum* infections diagnosed by RDTs and treated correctly or incorrectly.

Missing data were handled using multiple imputation by chained equations (50 imputations). Imputed data were used for the bivariate and multivariable GEE analyses; observed data were used for descriptive summaries and sensitivity analyses. Analyses were conducted in R (version 4.3.3) (Supplementary Table 1).

### Ethical Approval

This study was approved by the Institutional Review Boards of the University of North Carolina at Chapel Hill (IRB # 14-0489, 17-1588, and 24-0252) and the Kinshasa School of Public Health.

## RESULTS

### Study Population

Between February 2015 and December 2022, 2269 participants were enrolled across 1 urban (n = 622), 3 peri-urban (n = 808), and 3 rural (n = 839) sites (Table 1). These comprised 1592 and 1635 participants enrolled during Phase 1 and Phase 2, respectively, including 958 participants enrolled in both phases. Participants were mostly female (55%) with a median age of 14 years (interquartile range, IQR: 6–30). Participants completed 24 159 visits, including 9020 (37.3%) clinic visits. Household and clinic visits primarily captured asymptomatic and symptomatic infections, respectively (17.1% vs 71.5% infections with fever). On average, participants attended 16 study visits (median: 14, IQR: 10–20). Most participants had at least 1 clinic visit (n = 1714). During Phase 1 and Phase 2, 77% and 46% of participants completed all their household visits, respectively. No household visits were conducted at the urban site between March and April 2020 due to COVID-19 pandemic restrictions in that area; 71% of the participants in Phase 2 completed at least 6 of the 7 household visits.

**Table 1.** Baseline Characteristics of the Study Population by Study Area Reflecting High, Moderate, and Low Malaria Transmission Intensity Levels

	Rural	Peri-urban	Urban	Overall
	(N = 839)	(N = 808)	(N = 622)	(N = 2269)
Sex				
Male	382 (45.5%)	375 (46.4%)	264 (42.4%)	1021 (45.0%)
Female	457 (54.5%)	433 (53.6%)	358 (57.6%)	1248 (55.0%)
Age category (years)				
Under 5	237 (28.2%)	164 (20.3%)	71 (11.4%)	472 (20.8%)
5–14	248 (29.6%)	267 (33.0%)	163 (26.2%)	678 (29.9%)
15+	354 (42.2%)	377 (46.7%)	388 (62.4%)	1119 (49.3%)
Education level				
No education	76 (9.1%)	58 (7.2%)	11 (1.8%)	145 (6.4%)
Not applicable^a^	236 (28.1%)	162 (20.0%)	71 (11.4%)	469 (20.7%)
Primary school	299 (35.6%)	261 (32.3%)	128 (20.6%)	688 (30.3%)
Secondary school or other	209 (24.9%)	284 (35.1%)	292 (46.9%)	785 (34.6%)
University or higher	8 (1.0%)	9 (1.1%)	91 (14.6%)	108 (4.8%)
Missing	11 (1.3%)	34 (4.2%)	29 (4.7%)	74 (3.3%)
Agriculture work				
Non-farmer	254 (30.3%)	323 (40.0%)	366 (58.8%)	943 (41.6%)
Not applicable^a^	496 (59.1%)	442 (54.7%)	240 (38.6%)	1178 (51.9%)
Farmer	83 (9.9%)	25 (3.1%)	1 (0.2%)	109 (4.8%)
Missing	6 (0.7%)	18 (2.2%)	15 (2.4%)	39 (1.7%)
Wealth				
Poorest	244 (29.1%)	165 (20.4%)	0 (0%)	409 (18.0%)
Poorer	277 (33.0%)	166 (20.5%)	0 (0%)	443 (19.5%)
Average	229 (27.3%)	222 (27.5%)	0 (0%)	451 (19.9%)
Richer	89 (10.6%)	251 (31.1%)	110 (17.7%)	450 (19.8%)
Richest	0 (0%)	4 (0.5%)	512 (82.3%)	516 (22.7%)
Malaria (past 6 months)	231 (27.5%)	213 (26.4%)	169 (27.2%)	613 (27.0%)
Antimalarial treatment (past 6 months)	198 (23.6%)	220 (27.2%)	196 (31.5%)	614 (27.1%)
Fever (now/past 7 days)	277 (33.0%)	135 (16.7%)	67 (10.8%)	479 (21.1%)
*P. falciparum* by qPCR	370 (44.1%)	334 (41.3%)	25 (4.0%)	729 (32.1%)
*P. falciparum* by RDT	324 (38.6%)	254 (31.4%)	12 (1.9%)	590 (26.0%)

^a^Children under 5 years old for whom education was not indicated as “Primary school” were categorized as “Not applicable.” Employment status of children under 15 years of age was recorded as “Not applicable” if none were indicated.

### Malaria Burden

**Figure 2.**
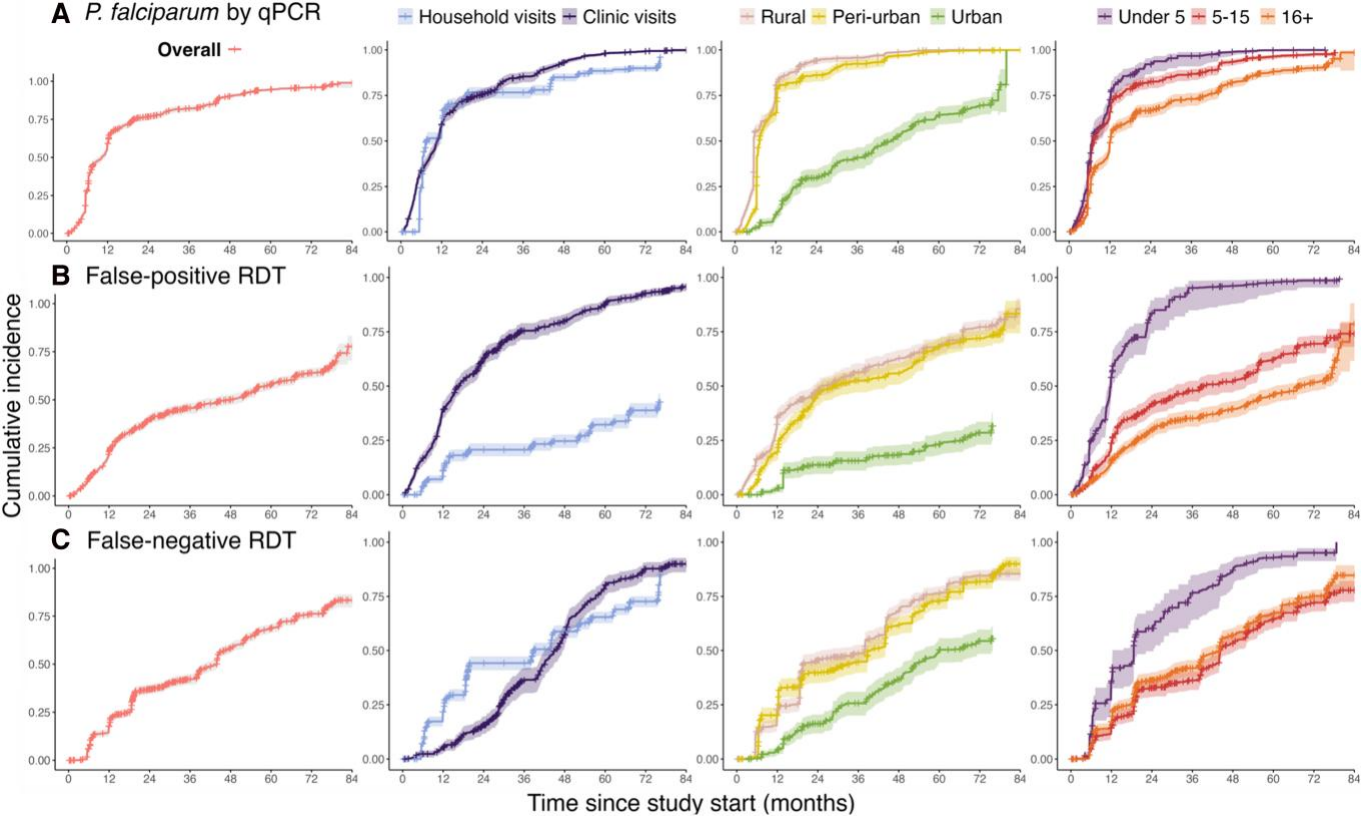
Cumulative incidence of (*A*) *P. falciparum* (qPCR), (*B*) false-positive rapid diagnostic test (RDT), and (*C*) false-negative RDT over the study period, overall and stratified by endemicity, surveillance, and age. Shaded areas around each curve represent the 95% CIs. Abbreviations: CIs, confidence intervals; qPCR, quantitative polymerase chain reaction.

The 1-year cumulative incidence of a first *P. falciparum* infection by qPCR was 58.9% (95% CI, CI: 56.3–61.3%), rising to 99.2% (95% CI: 94.3–99.9%) by the end of the study (Figure 2; Supplementary Tables 2 and 3). The highest cumulative incidence was observed in the rural and peri-urban sites. qPCR and RDT data were missing for 6% and 8% of the 24 159 visits, respectively. In the observed data, of all samples collected during household visits over 7 years, *P. falciparum* was detected in 41.4% by qPCR (5947/14 378) and 32.6% by RDT (4772/14 648). The proportion of clinic samples positive for *P. falciparum* was 67.6% by qPCR (5621/8318) and 77.0% by RDT (5877/7630). Variations in the proportion of *P. falciparum* detected by household follow-up and clinic period are shown in Supplementary Figure 2. Malaria burden was highest in the rural sites (63.1% by qPCR and 61.5% by RDT) and lowest in the urban site (14.7% by qPCR and 9.7% by RDT) (Supplementary Table 4).

### Rapid Diagnostic Test Diagnostic Performance

Using qPCR as the gold standard and focusing on samples with both qPCR and RDT results available (n = 20 881), 40.6% of RDTs were true-negative, 38.1% true-positive, 11.8% false-negative, and 9.4% false-positive (Table 2). Agreement between qPCR and RDT *P. falciparum* results was moderate (Cohen's Kappa = 0.58; *P*-value < .05). Rapid diagnostic test sensitivity was 76.3% and specificity 81.2%. As expected, positive predictive value and negative predictive value varied with transmission intensity (Supplementary Table 4). Positive predictive value was highest in the peri-urban sites and lowest in the urban site (81.9% vs 73.1%). Negative predictive value was highest in the urban site and lowest in the rural sites (92.8% vs 65.2%).

**Table 2.** Study Population Characteristics Stratified by *P. falciparum* Rapid Diagnostic Test (RDT) Results Using Quantitative Polymerase Chain Reaction (qPCR) as the Gold Standard

	qPCR Negative		qPCR Positive	
	RDT Negative	RDT Positive	RDT Negative	RDT Positive
	(True-Negative RDT)	(False-Positive RDT)	(False-Negative RDT)	(True-Positive RDT)
	(N = 8479)	(N = 1969)	(N = 2468)	(N = 7965)
Sex				
Male	3617 (42.7%)	846 (43.0%)	1134 (45.9%)	3764 (47.3%)
Female	4862 (57.3%)	1123 (57.0%)	1334 (54.1%)	4201 (52.7%)
Age category (years)				
Under 5	1217 (14.4%)	429 (21.8%)	262 (10.6%)	1340 (16.8%)
5–15	2395 (28.2%)	781 (39.7%)	798 (32.3%)	4294 (53.9%)
16+	4867 (57.4%)	759 (38.5%)	1408 (57.1%)	2331 (29.3%)
Wealth				
Poorest	1150 (13.6%)	436 (22.1%)	545 (22.1%)	2112 (26.5%)
Poorer	1225 (14.4%)	483 (24.5%)	565 (22.9%)	1923 (24.1%)
Average	1400 (16.5%)	579 (29.4%)	606 (24.6%)	2002 (25.1%)
Richer	1767 (20.8%)	371 (18.8%)	527 (21.4%)	1688 (21.2%)
Richest	2937 (34.6%)	100 (5.1%)	225 (9.1%)	240 (3.0%)
Fever (now/past 7 days)^a^	994 (11.7%)	847 (43.0%)	540 (21.9%)	3385 (42.5%)
Treatment (now/past 7 days)^b^	481 (5.7%)	1248 (63.4%)	389 (15.8%)	3911 (49.1%)
RDT brand				
Pf HRP2/Pan-LDH	5436 (64.1%)	1352 (68.7%)	1471 (59.6%)	4656 (58.5%)
Pf HRP2 only	3043 (35.9%)	617 (31.3%)	997 (40.4%)	3309 (41.5%)
Surveillance arm				
Household visits	7507 (88.5%)	683 (34.7%)	1864 (75.5%)	3851 (48.3%)
Clinic visits	972 (11.5%)	1286 (65.3%)	604 (24.5%)	4114 (51.7%)
Season				
Dry season	2379 (28.1%)	573 (29.1%)	740 (30.0%)	2124 (26.7%)
Rainy season	6100 (71.9%)	1396 (70.9%)	1728 (70.0%)	5841 (73.3%)
Health area				
Rural	2264 (26.7%)	1137 (57.7%)	1210 (49.0%)	4393 (55.2%)
Peri-urban	2727 (32.2%)	728 (37.0%)	988 (40.0%)	3289 (41.3%)
Urban	3488 (41.1%)	104 (5.3%)	270 (10.9%)	283 (3.6%)
*P. falciparum* in past month^c^	228 (40.9%)	294 (66.4%)	104 (54.2%)	731 (66.6%)
ACT in past month^c^	320 (57.5%)	291 (65.7%)	106 (55.2%)	600 (54.6%)
*P. falciparum* density				
Under 200/μL	NA	NA	2161 (87.6%)	4032 (50.6%)
At least 200/μL	NA	NA	307 (12.4%)	3933 (49.4%)
*P. falciparum*/μL (log10)				
Mean (SD)	NA	NA	0.98 (1.11)	2.30 (1.31)

Abbreviations: ACT, artemisinin-based combination therapy; NA, not applicable; SD, standard deviation.

^a^During the household visits, fever denotes self-reported fever in the 7 days preceding the household visit. During clinic visits, fever denotes current fever, either self-reported or measured by a clinic staff using a thermometer.

^b^Data on antimalarial treatment were primarily collected during the clinic visits and refer to whether a clinic staff provided treatment to a participant.

^c^Data on *P. falciparum* infection by qPCR and ACT use in the past month were restricted to the few participants who had 2 visits within 30 days of each other.

### Extent and Correlates of False-Positive and False-Negative Rapid Diagnostic Tests

**Figure 3.**
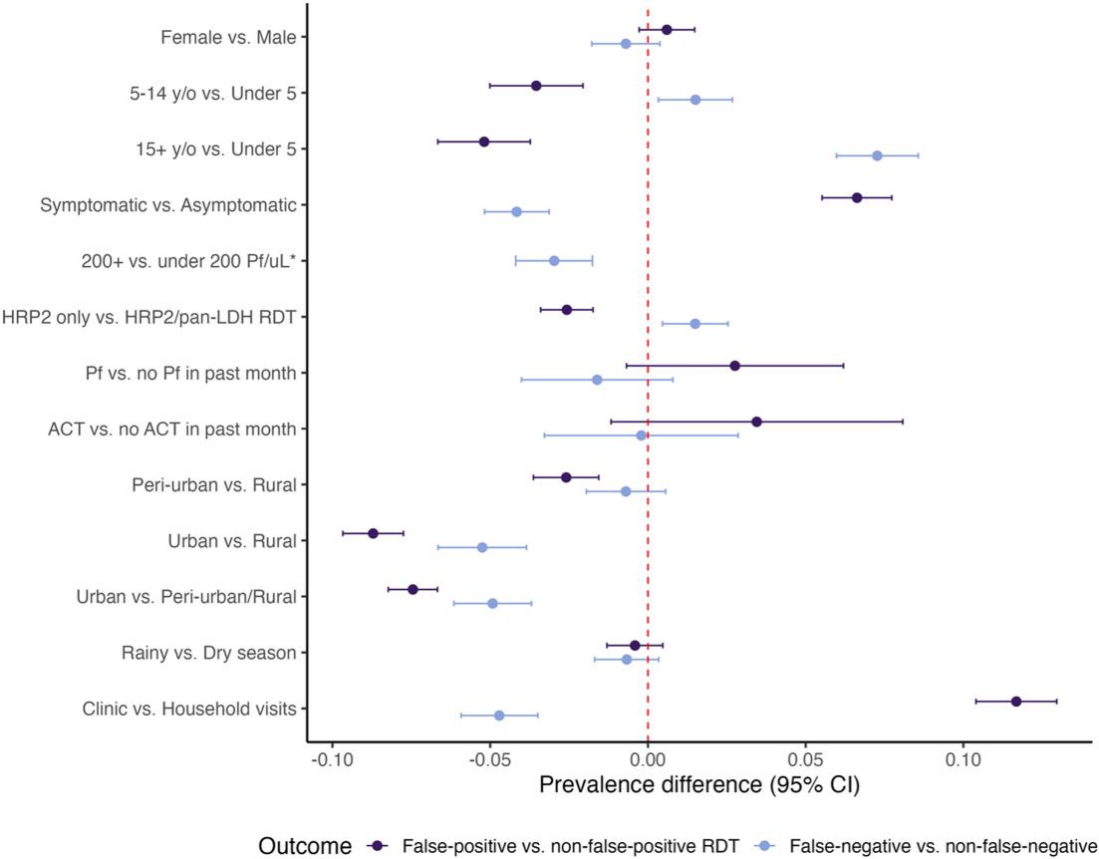
Correlates of false-positive and false-negative *P. falciparum* rapid diagnostic test (RDT) results. False-positive RDTs were defined as RDT-positive but qPCR-negative, and false-negative RDTs were defined as RDT-negative but qPCR-positive (dots = point estimates; horizontal bar around each dot = 95% CI; dotted red line = null value). *Parasite densities are absent in the setting of false-positive and true-negative RDT results and therefore cannot be modeled. Abbreviation: qPCR, quantative polymerase chain reaction.

The cumulative incidence of a first false-positive RDT was 23.1% (95% CI: 20.8–25.3%) at 1 year and 77.7% (95% CI: 70.2–83.3%) at 7 years (Figure 2). False-positive RDTs were more common during clinic visits (range: 10.6%–28.4%) than household visits (1.9%–8.5%). Malaria was more likely to be overdiagnosed during visits where participants were younger (proportion difference [PD] of false-positive RDT = −3.5% [95% CI: −5.0 to −2.1%] among school-aged children and PD = −5.2% [95% CI: −6.7 to −3.7%] among those 15 + compared to those under 5), were symptomatic (PD = 6.6%, 95% CI: 5.5–7.7%), had a recent falciparum infection (PD = 2.8%, 95% CI: −0.7% to 6.2%), and received treatment in the preceding month (PD = 3.5%, 95% CI: −1.2% to 8.1%). The proportion of false-positive RDTs increased with malaria transmission intensity and was lower during the study period when HRP2-only RDTs were used (Figure 3 and Table 2). Results from complete-case analyses are shown in Supplementary Figure 3.

The cumulative incidence of a first false-negative RDT was 17.8% (95% CI: 15.8–19.8%) at 1 year and 83.4% (95% CI: 79.7–86.4%) at 7 years (Figure 2). False-negative RDTs followed the opposite pattern of false-positive RDTs, being more common during household visits (8.1% to 19.8%) than clinic visits (1.0% to 19.0%). Infections in older participants harboring low-density infections were more likely to be missed by RDTs (Figure 3 and Table 2). False-negative RDTs were negatively associated with transmission intensity. Infections with density under 200 parasites/μL were more likely to yield false-negative RDTs (PD = 3.0%, 95% CI: 1.8–4.2%), as expected.

Overall, analyses investigating correlates of RDT sensitivity and specificity showed similar results, with some differences highlighted in Table 2 and Supplementary Figure 4.

### Extent and Correlates of Inappropriate Treatment

Nearly all participants presenting to clinics received testing by malaria RDT; RDT was not performed during 17 of the 9020 clinic visits. Data on antimalarial treatment and RDT results were missing for 15% and 20% of the clinic visits, respectively. Treatment was prescribed during 47.1% of visits during which RDT was not performed (8/17) and 58.1% of visits when participants were RDT-negative (755/1299) (Table 3). Overtreatment was highly prevalent among RDT-negative participants with fever (83.2%, 307/369 visits). Among the 5907 visits by RDT-positive participants with treatment data, undertreatment was rare; participants were not treated at 3.7% of these visits (n = 217).

**Table 3.** Overall and Symptom-Stratified Crude Proportion of Antimalarial Treatment by Rapid Diagnostic Test (RDT) Results Among Study Participants Presenting to Health Facilities at the Study Sites Compared to a Hypothetical Population With Perfect Adherence to Treatment Guidelines

	No Treatment	Treatment	N	Proportion Treated	Crude PD^a^ (95% CI)
Hypothetical perfect adherence (N = 7206 visits)					
RDT negative	1299	0	1299	0.0%	100.0%
RDT positive	0	5907	5907	100.0%	
All participants (N = 7206 visits)^b^					
RDT negative	544	755	1299	58.1%	38.2% (35.5–40.9)
RDT positive	217	5690	5907	96.3%	
Afebrile and febrile participants (N = 6011 visits) ^c^					
RDT negative	208	627	835	75.1%	21.1% (18.1–24.1)
RDT positive	197	4979	5176	96.2%	
Afebrile participants (N = 1909 visits)					
RDT negative	146	320	466	68.7%	25.4% (21.0–29.8)
RDT positive	86	1357	1443	94.0%	
Febrile participants (N = 4102 visits)					
RDT negative	62	307	369	83.2%	13.8% (10.0–17.7)
RDT positive	111	3622	3733	97.0%	

^a^PD, proportion difference; 95% CI, 95% confidence interval.

^b^Fever, rapid diagnostic test results, and treatment data were missing for some of the 9020 clinic visits recorded.

^c^The number of afebrile + febrile participants is different from that of all participants because some of the participants were missing data on fever.

Overtreatment was more common at clinic visits when participants were symptomatic (PD = 18.0%, 95% CI: 8.4–27.5%), treated during a prior clinic visit (PD = 26.5%, 95% CI: 20.4–32.6%), and residents in the peri-urban or rural sites (PD = 10.6%, 95% CI: 6.9–20.5%). More overtreatment was observed during the COVID-19 pandemic (PD = 16.2%, 95% CI: 4.3–28.0%). Although undertreatment was rare, some significant correlates were identified, with the association in the opposite direction of overtreatment for most correlates. Both overtreatment and undertreatment were more prevalent at the peri-urban sites, suggesting poorer adherence at these sites. Supplementary Figure 5 lists the factors associated with adherence to treatment guidelines.

### Rapid Diagnostic Test Results and Treatment Decisions

If there were complete adherence to treatment guidelines, all RDT-positive and no RDT-negative participants would receive treatment, regardless of symptoms. This would result in a treatment proportion difference of 100% comparing RDT-positive to RDT-negative results (Tables 3; Supplementary Table 5). In this study population, the estimated proportion difference was much lower than expected, with a weighted difference of 27.5% (95% CI: 26.9–28.1%). Non-adherence was primarily driven by overtreatment of RDT-negative participants. Moreover, the presence of fever significantly influenced adherence (*P*-value < .0001). Adherence was higher among asymptomatic participants (32.9%, 95% CI: 32.1–33.7%) than symptomatic participants (22.7%, 95% CI: 21.9–23.5%).

### From Infection to Diagnosis and Treatment

**Figure 4.**
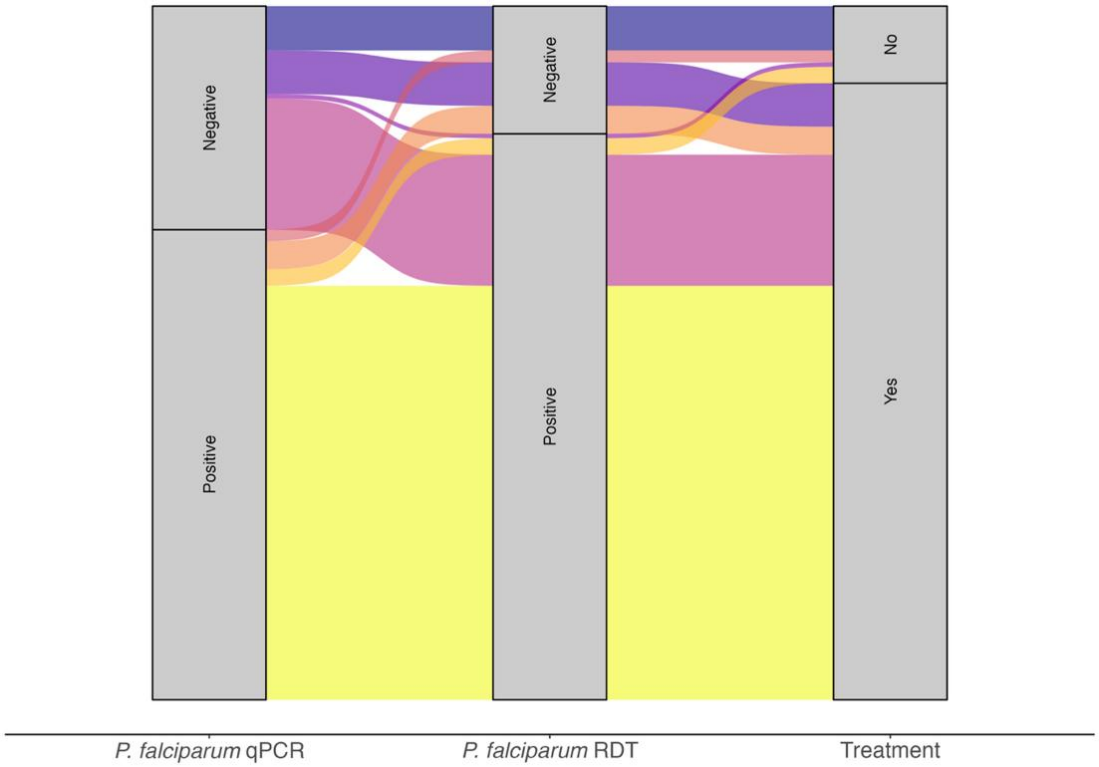
Cascade from *P. falciparum* infection confirmed by qPCR, diagnosis in the field by rapid diagnostic test (RDT), and treatment during the clinic visits. Non-falciparum *Plasmodium* RDT results (HRP2-negative, pan-LDH-positive) are not included in this analysis.

The proportion of untreated qPCR-confirmed falciparum infections was low (6.2%, 279/4535); however, treatment was prescribed at 78.4% of visits when participants were *P. falciparum*-negative (1667/2126). Most qPCR-confirmed falciparum infections were correctly diagnosed by RDT and treated while a small proportion was incorrectly diagnosed yet still treated. About half of participants negative by both qPCR and RDT still received treatment (Figure 4).

## DISCUSSION

Most participants experienced at least 1 malaria misdiagnosis over the 7-year study period. We identified specific factors—such as age, recent treatment, transmission intensity, and parasite density—that influenced RDT accuracy and could inform refinements to treatment guidelines. These findings underscore persistent challenges in malaria case management in high-burden settings like the DRC, where over 99% of participants had an incident infection during the study period. Non-adherence to test-and-treat policies, particularly among RDT-negative febrile participants, was widespread. This practice was probably driven by low confidence in negative results and limited diagnostic alternatives. Findings from this study highlight opportunities to improve clinical decision-making and enhance the effectiveness of malaria test-and-treat strategies.

Presumptive treatment was pervasive, especially among symptomatic individuals in rural and peri-urban sites. This is consistent with findings across SSA showing overprescription of antimalarials at some health facilities, including reports that 50%–83% of RDT-negative symptomatic individuals in highly endemic areas still received treatment [12, 13, 27–29]. Factors cited by providers to justify overtreatment include low confidence in RDT-negative results, potential for false-negative RDTs among individuals who started treatment prior to presentation, and patients' expectation to be treated for malaria regardless of RDT results [27, 30, 31].

False-positive RDTs were common in the clinical setting, particularly among younger participants and marginally among those recently treated. These factors should be considered by clinicians to improve diagnostic accuracy. Frequent reinfections in high-transmission settings likely lead to persistent HPR2 antigenemia despite successful treatment and could explain the higher prevalence of false-positive RDTs noted in rural and peri-urban areas. This finding aligns with reports that false-positive RDT prevalence and overall RDT performance vary with malaria transmission intensity [6,7, 12, 17]. The high prevalence of false-negative RDTs in a community setting where we recently found no *pfhrp2* deletion highlights the limitations of conventional malaria RDTs for the detection of asymptomatic, low-density infections [17, 20]. The differential performance of RDTs in clinical and community settings has important implications for malaria control, with malaria overdiagnosed and other etiologies of febrile illness overlooked during clinic visits, and asymptomatic infections undetected and contributing to ongoing transmission in the community setting.

Nearly all RDT-positive participants appropriately received treatment, though 18% of these RDTs were false-positive. While false-negative RDTs did occur in the population, they were primarily among asymptomatic adults with low parasite density and therefore at lower risk of malaria morbidity and mortality. Foregoing treatment guidelines to treat these few RDT-negative individuals is difficult to justify in the study area, though a balance between the need to decrease the malaria transmission reservoir and prevent drug emergence may be of higher significance in other settings. Treatment of febrile, RDT-negative participants was common, a finding likely driven by constraints of limited diagnostic and treatment options for non-malarial febrile illness in the DRC.

This study has limitations. First, antimalarial overuse was likely underestimated as analyses were limited to the clinical setting. At-home treatment is common in the DRC, where self-medication often involves monotherapies that are no longer recommended [32]. Second, we used provider antimalarial prescription as a proxy for treatment. Failure to complete self- or provider-prescribed regimens is well documented in SSA and may contribute to drug resistance [33]. Third, our analysis of symptoms focused on fever. It is possible that other clinical symptoms may further influence guideline adherence. Despite these limitations, the inclusion of sites with varied transmission intensities captured malaria spatial heterogeneity and strengthens the generalizability of the findings in high-burden settings like the DRC.

This study reaffirms the heavy burden of *P. falciparum* in the DRC and highlights opportunities for improved management of malaria cases. Clinicians should review recent treatment history to avoid overtreatment of patients with lingering antigenemia and refer for microscopy to confirm diagnosis when in doubt. More comprehensive diagnostic options are needed to guide care of RDT-negative patients and avoid presumptive treatment. Current national guidelines require parasitological confirmation by RDT or microscopy before initiating treatment to avoid unnecessary drug pressure and resistance and emphasize the need to investigate non-malarial causes of fever when malaria tests are negative [34]. Reinforcing adherence to test-and-treat policies and improving management of non-malarial febrile illness are essential to reduce overtreatment, preserve drug efficacy, and improve quality of care in the DRC and similar settings.

## Supplementary Material

ciag262_Supplementary_Data

## Supplementary Data

Supplementary materials are available at *Clinical Infectious Diseases* online. Consisting of data provided by the authors to benefit the reader, the posted materials are not copyedited and are the sole responsibility of the authors, so questions or comments should be addressed to the corresponding author.
