# Supplementary material for: Challenges in Malaria Diagnosis and Treatment in Kinshasa Province, Democratic Republic of the Congo
Source: Clin Infect Dis. 2026 Apr 18;83(1):e196–205. doi: 10.1093/cid/ciag262 (PMC13393125; doi:10.1093/cid/ciag262)
Supplement: ciag262_Supplementary_Data [file ciag262_supplementary_data.zip › Francois-Zafka_Manuscript 1_draft 11_supp_RFZ_KLM.docx]

**SUPPLEMENTARY MATERIALS**

1. **SUPPLEMENTARY METHODS**

**A1. Study enrollment across Phase 1 and Phase 2**

Enrollment for Phase 1 occurred between February and June 2015; household visits during this phase lasted until October 2016 while clinic visits continued until January 2018. During Phase 1, a baseline and three household follow-up visits were conducted (four scheduled household visits total). At the end of Phase 1, participants were invited to participate in Phase 2 and re-enrolled upon consent. If a household refused to continue in the study, a nearby household was recruited. Enrollment for Phase 2 occurred primarily between March and April 2018, and between September and October 2018 for a small subset of the participants (n = 31). Household visits were conducted until May 2021, and clinic visits until December 2022. During Phase 2, a baseline and six household follow-up visits were conducted (seven scheduled household visits total).

**A2. Survival analysis**

Time since study start was chosen as the timescale, in lieu of time on study, to account for the two study phases, late enrollment during Phase 2, prevalent or missing outcome at first visit, potential seasonal variation, change in RDT brand in 2019, and COVID-19 pandemic in March 2020.^24,35^ Entry time was estimated as the number of months between the study start date (February 1^st^, 2015) and the date when participants entered the risk set. Participants without the outcome at their first visit entered the risk-set at their first visit; those with the outcome present or unknown at their first visit entered the risk set 30 days later to allow for infection and antigenemia clearing.^8,9^ Exit time was defined as the number of months from the start of the study to outcome or censoring. Participants were removed from the risk set once they experienced their first outcome. Participants lost to follow-up were censored at their last visit.^36^ Sex, baseline age, wealth, health area, and entry time were included in the models to derive the stabilized inverse probability censoring weights used to correct Kaplan-Meier curves for informative censoring.^35,37^

**A3. Factors**

Key demographic, clinical, community, and parasite characteristics were investigated as potential correlates of malaria misdiagnosis and inappropriate treatment based on literature review.

**Demographic factors**: Sex was defined as a binary variable (male, female) to explore its association with misdiagnosis considering reports of faster clearance of asymptomatic infections by females compared to males, and its association with inappropriate treatment considering potential gender bias in care delivery.^1,38^ Age was treated as a categorical variable (children under 5 years old, school-aged children 5 to 15 years old, and adults 16 years or older). These categories were selected as relevant to malaria epidemiology given the highest burden among children under 5 years old, the increasingly recognized role of school-aged children as an important malaria transmission reservoir, the acquired immunity with age allowing adults in endemic areas to remain asymptomatic despite relatively high parasite density, and the high prevalence of overtreatment among children.^12,39^ Education level was a categorical level for which data were collected for anyone at least 5 years old (no education, primary school, secondary school or other, higher education). Children under 5 years old for whom education was not indicated as “primary school” were categorized as “not applicable.” Data on over 16 occupations were collected from participants at least 15 years old and dichotomized as “non-farmer” or “farmer.” Employment status of children under 15 years of age was recorded as “not applicable” if none were indicated. The “not applicable” category for education and employment was created to avoid missingness and to reflect the study approach to these questions. Household wealth is associated with lower malaria and was created using the Demographic and Health Survey method to determine wealth quintiles (poorest, poorer, average, richer, Richest) based on baseline household characteristics and ownership of different possessions.^24,40^ The wealth variable was recategorized as a three-level variables for some analyses (poorest/poorer, average, richer/richest) to minimize non-positivity because only the richer and richest participants were found in the urban site, and none of the richest participants were found in the rural sites. Participants in the same household were attributed the same wealth quintile regardless of their education or employment status.

**Malaria-related factors**: During the household visits, participants were asked about self-reported malaria-related factors experienced during the 6 months preceding a follow-up visit: diagnosis (a binary yes/no variable and a categorical variable as none, once, at least twice), symptoms (a non-mutually exclusive list including malaria symptoms, but recategorized as yes/no), and treatment (a list of antimalarial drugs that were recategorized as yes/no. During the household and clinic visits, current fever or fever in the previous week was recorded as either self-reported or measured with a thermometer by a clinic staff. During clinic visits, treatment prescription for the current or recent fever was recorded as yes/no and the drug prescribed was also reported.

We also derived variables from those collected from the questionnaires. Given reports of persistent HRP2 antigenemia and the association between persistence and recent ACT treatment, we created a binary variable to indicate whether a falciparum infection was detected by qPCR in the preceding 30 days, and another binary variable to indicate if any antimalarial treatment was administered in the preceding 30 days. For analyses examining the association between recent infection (or recent treatment) and RDT result, we only considered infection and treatment within the preceding 30 days and restricted analyses to participants who had a visit in the preceding 30 days.

We created a binary variable (yes/no) to reflect whether participants had received treatment during the prior clinic visits to capture if they were more likely to receive treatment based on prior experience. Last, we created a variable to approximate malaria history to-date as the number of RDT-positive results and self-reported malaria diagnoses; this definition was chosen as a proxy of participants’ perception of susceptibility to malaria.

**Study-related factors**: During the study, there was a change in RDT used; given the difference in sensitivity and specificity of RDT brands, a binary variable was created to indicate RDT brand. Also noteworthy was the COVID-19 pandemic, which may have affected participants’ and health professionals’ attitude towards febrile illnesses. A binary COVID-19 pandemic was created and defined as pre-COVID (from Phase 1 to Phase 2 follow-up 3) and COVID/post-COVID (from Phase 2 follow-up 4 to the end of the study).

1. **SUPPLEMENTARY TABLES AND FIGURES**

**Table S1.** List of key R packages used for analyses

| **Purpose** | **Package** | **Version** | **Citation** |
| --- | --- | --- | --- |
| Data wrangling | tidyverse | 2.0.0 | Wickham H, Averick M, Bryan J, Chang W, McGowan LD, François R, Grolemund G, Hayes A, Henry L, Hester J, Kuhn M, Pedersen TL, Miller E, Bache SM, Müller K, Ooms J, Robinson D, Seidel DP, Spinu V, Takahashi K, Vaughan D, Wilke C, Woo K, Yutani H (2019). “Welcome to the tidyverse.” _Journal of Open Source Software, *4*(43), 1686. doi:10.21105/joss.01686 <https://doi.org/10.21105/joss.01686>. |
| Cohen’s Kappa statistic | irr | 0.84.1 | Gamer M, Lemon J, <puspendra.pusp22@gmail.com> IFPS (2019). _irr: Various Coefficients of Interrater Reliability and Agreement_. R package version 0.84.1, <https://CRAN.R-project.org/package=irr>. |
| Multiple imputation | mice | 3.16.0 | Stef van Buuren, Karin Groothuis-Oudshoorn (2011). mice: Multivariate Imputation by Chained Equations in R. Journal of Statistical Software, 45(3), 1-67. DOI 10.18637/jss.v045.i03. |
| Generalized Estimating Equations (GEE) | geepack | 1.3.12 | Højsgaard, S., Halekoh, U. & Yan J. (2006) The R Package geepack for Generalized Estimating Equations Journal of Statistical Software, 15, 2, pp1--11  Yan, J. & Fine, J.P. (2004) Estimating Equations for Association Structures Statistics in Medicine, 23, pp859--880.  Yan, J (2002) geepack: Yet Another Package for Generalized Estimating Equations R-News, 2/3, pp12-14. |
| Model estimate pooling | mitools | 2.4 | Lumley T (2019). _mitools: Tools for Multiple Imputation of Missing Data. R package version 2.4,  <https://CRAN.R-project.org/package=mitools>. |
| Survival analysis | survival | 3.5.8 | Therneau T (2024). _A Package for Survival Analysis in R. R package version 3.5-8, <https://CRAN.R-project.org/package=survival>.  Terry M. Therneau, Patricia M. Grambsch (2000). Modeling Survival Data: Extending the Cox Model. Springer, New York. ISBN 0-387-98784-3. |
|  | survminer | 0.5.0 | Kassambara A, Kosinski M, Biecek P (2024). _survminer: Drawing Survival Curves using 'ggplot2'. R package version 0.5.0, <https://CRAN.R-project.org/package=survminer>. |
| Parallel processing when bootstrapping | furrr | 0.3.1 | Vaughan D, Dancho M (2022). _furrr: Apply Mapping Functions in Parallel using Futures. R package version 0.3.1, <https://CRAN.R-project.org/package=furrr>. |
|  | future | 1.40.0 | Henrik Bengtsson, A Unifying Framework for Parallel and Distributed Processing in R using Futures, The R Journal (2021) 13:2, pages 208-227, doi:10.32614/RJ-2021-048 |
|  | future.apply | 1.11.3 | Henrik Bengtsson, A Unifying Framework for Parallel and Distributed Processing in R using Futures, The R Journal (2021) 13:2, pages 208-227, doi:10.32614/RJ-2021-048 |

**Table S2.**  Cumulative incidences of the falciparum infection, false-positive rapid diagnostic test (RDT), and false-negative RDT over the seven-year study period

|  | ***P. falciparum* (qPCR)** | | | **False-positive RDT** | | | **False-negative RDT** | | |
| --- | --- | --- | --- | --- | --- | --- | --- | --- | --- |
| **Time (months)** | **# at risk** | **# of events** | **Risk, % (95% CI)** | **# at risk** | **# of events** | **Risk, % (95% CI)** | **# at risk** | **# of events** | **Risk, % (95% CI)** |
| 0 | 1569 | 0 | 0 | 1438 | 0 | 0 | 1465 | 0 | 0 |
| 6 | 1098 | 438 | 28.5 (26.2-30.7) | 1264 | 129 | 9.2 (7.7-10.7) | 1330 | 85 | 6 (4.7-7.2) |
| 12 | 596 | 448 | 58.9 (56.3-61.3) | 1004 | 183 | 23.1 (20.8-25.3) | 1103 | 162 | 17.8 (15.8-19.8) |
| 18 | 414 | 182 | 71.2 (68.8-73.4) | 864 | 132 | 33.1 (30.5-35.6) | 996 | 100 | 25.1 (22.7-27.3) |
| 24 | 260 | 68 | 76.3 (74-78.4) | 620 | 76 | 40 (37.2-42.6) | 698 | 153 | 37 (34.3-39.6) |
| 30 | 287 | 53 | 79.9 (77.6-82) | 719 | 48 | 43.6 (40.7-46.3) | 762 | 41 | 40.1 (37.3-42.8) |
| 36 | 273 | 23 | 81.4 (79.2-83.5) | 701 | 29 | 45.8 (42.8-48.5) | 745 | 27 | 42.2 (39.3-44.9) |
| 42* | 752 | 127 | 84.1 (82.1-85.9) | 1182 | 53 | 48.1 (45.2-50.8) | 1159 | 152 | 48.8 (46-51.5) |
| 48 | 503 | 254 | 89.4 (87.9-90.7) | 1147 | 47 | 50.1 (47.2-52.8) | 945 | 218 | 58.3 (55.7-60.8) |
| 54 | 355 | 133 | 92.2 (91-93.2) | 1045 | 86 | 53.8 (51-56.4) | 785 | 140 | 64.4 (61.9-66.8) |
| 60 | 275 | 75 | 93.8 (92.7-94.7) | 941 | 96 | 57.9 (55.2-60.5) | 683 | 99 | 68.8 (66.4-71) |
| 66 | 272 | 30 | 94.4 (93.4-95.2) | 919 | 53 | 60.2 (57.5-62.7) | 626 | 82 | 72.4 (70.0-74.5) |
| 72 | 216 | 35 | 95.2 (94.2-95.9) | 746 | 86 | 64.1 (61.4-66.5) | 494 | 83 | 76.2 (73.9-78.2) |
| 78 | 11 | 21 | 97.5 (95.5-98.6) | 50 | 28 | 67.9 (64.2-71.2) | 46 | 34 | 81.5 (78.3-84.3) |
| 84 | 1 | 3 | 99.2 (94.3-99.9) | 18 | 11 | 77.7 (70.2-83.3) | 20 | 4 | 83.4 (79.7-86.4) |
| Cumulative incidences were estimated using Kaplan-Meier curves weighted to account for informative censoring. The number at risk and number of events were rounded to the nearest whole number.  * The increase in the number of at risk represents susceptible participants newly enrolled approximately 3 years after the study start (Phase 2 enrollees). | | | | | | | | | |

**Table S3. One- and eight-year cumulative incidences of first *P. falciparum* infection by qPCR, overall and by health area**

|  | **Overall (95% CI)** | **Rural (95% CI)** | **Peri-urban (95% CI)** | **Urban (95% CI)** |
| --- | --- | --- | --- | --- |
| **1-year** | 58.86% (56.30 - 61.27) | 80.68% (77.41 - 83.50) | 65.50% (61.10 - 69.40) | 9.50% (6.30 - 12.50) |
| **8-year** | 99.25% (94.31 - 99.90) | 99.97% (99.76 - 100.00) | 99.90% (99.46 - 99.98) | 81.00% (65.90 - 89.40) |

**Table S4.** *P. falciparum* prevalence by qPCR and rapid diagnostic test (RDT) and RDT diagnostic performance, overall and by surveillance arm and health area (endemicity)

|  | **Overall (95% CI)** | **Rural health area (95% CI)** | **Peri-urban health area (95% CI)** | **Urban health area (95% CI)** |
| --- | --- | --- | --- | --- |
| ***Overall*** |  |  |  |  |
| Prevalence (qPCR) | 50.97% (50.32 - 51.62) | 63.14% (62.16 - 64.10) | 56.60% (55.54 - 57.65) | 14.66% (13.64 - 15.72) |
| Prevalence (RDT) | 47.80% (47.14 - 48.46) | 61.47% (60.49 - 62.44) | 52.63% (51.54 - 53.72) | 9.67% (8.82 - 10.58) |
| Sensitivity | 76.34% (75.52 - 77.16) | 78.40% (77.30 - 79.48) | 76.90% (75.61 - 78.16) | 51.18% (46.92 - 55.42) |
| Specificity | 81.15% (80.39 - 81.90) | 66.57% (64.95 - 68.15) | 78.93% (77.53 - 80.28) | 97.10% (96.50 - 97.63) |
| Positive predictive value | 80.18% (79.38 - 80.96) | 79.44% (78.35 - 80.50) | 81.88% (80.65 - 83.06) | 73.13% (68.41 - 77.48) |
| Negative predictive value | 77.46% (76.66 - 78.23) | 65.17% (63.56 - 66.76) | 73.41% (71.95 - 74.82) | 92.82% (91.94 - 93.62) |
| ***Household visits*** |  |  |  |  |
| Prevalence (qPCR) | 41.36% (40.56 - 42.17) | 56.98% (55.69 - 58.26) | 46.29% (44.95 - 47.64) | 5.47% (4.72 - 6.31) |
| Prevalence (RDT) | 32.58% (31.82 - 33.34) | 45.82% (44.56 - 47.09) | 35.47% (34.17 - 36.78) | 4.42% (3.75 - 5.17) |
| Sensitivity | 67.38% (66.15 - 68.60) | 69.36% (67.73 - 70.96) | 66.11% (64.15 - 68.03) | 47.65% (39.94 - 55.43) |
| Specificity | 91.66% (91.04 - 92.25) | 84.98% (83.50 - 86.37) | 90.75% (89.60 - 91.81) | 97.96% (97.39 - 98.44) |
| Positive predictive value | 84.94% (83.86 - 85.97) | 85.79% (84.38 - 87.11) | 85.91% (84.22 - 87.49) | 57.04% (48.47 - 65.31) |
| Negative predictive value | 80.11% (79.29 - 80.91) | 67.97% (66.29 - 69.62) | 75.84% (74.34 - 77.29) | 97.05% (96.39 - 97.63) |
| ***Clinic visits*** |  |  |  |  |
| Prevalence (qPCR) | 67.58% (66.56 - 68.58) | 72.49% (71.05 - 73.91) | 73.70% (72.14 - 75.22) | 37.80% (35.15 - 40.51) |
| Prevalence (RDT) | 77.02% (76.06 - 77.96) | 87.60% (86.48 - 88.66) | 83.61% (82.22 - 84.94) | 25.58% (23.04 - 28.25) |
| Sensitivity | 87.20% (86.21 - 88.14) | 90.46% (89.22 - 91.61) | 89.97% (88.54 - 91.27) | 52.74% (47.61 - 57.83) |
| Specificity | 43.05% (40.99 - 45.12) | 19.14% (16.68 - 21.78) | 33.15% (29.69 - 36.75) | 92.81% (90.44 - 94.75) |
| Positive predictive value | 76.19% (75.03 - 77.32) | 73.85% (72.22 - 75.43) | 78.59% (76.82 - 80.28) | 82.45% (77.10 - 87.00) |
| Negative predictive value | 61.68% (59.22 - 64.08) | 44.28% (39.41 - 49.23) | 54.78% (49.93 - 59.56) | 75.41% (72.13 - 78.48) |
| CI: Confidence Interval | | | | |

**Table S5.** Overall and symptom-stratified weighted proportion of antimalarial treatment by rapid diagnostic test (RDT) results among study participants presenting to health facilities at the study sites, comparing results obtained using complete vs. imputed data

|  | **PD (95% CI) – Complete-case** | **PD (95% CI) – Imputed data** |
| --- | --- | --- |
| **Overall** | 24.1% (19.8-28.4%) | 27.5% (26.9-28.1%) |
| **Afebrile participants** | 32.8% (25.7-39.9%) | 32.9% (32.1-33.7%) |
| **Febrile participants** | 17.1% (11.5-22.4%) | 22.7% (21.9-23.5%) |
| PD (95% CI): Proportion difference (95% confidence interval) | | |

**Figure S1.** Directed acyclic graph depicting the hypothesized relationship between malaria rapid diagnostic test result (exposure), treatment (outcome), and other factors


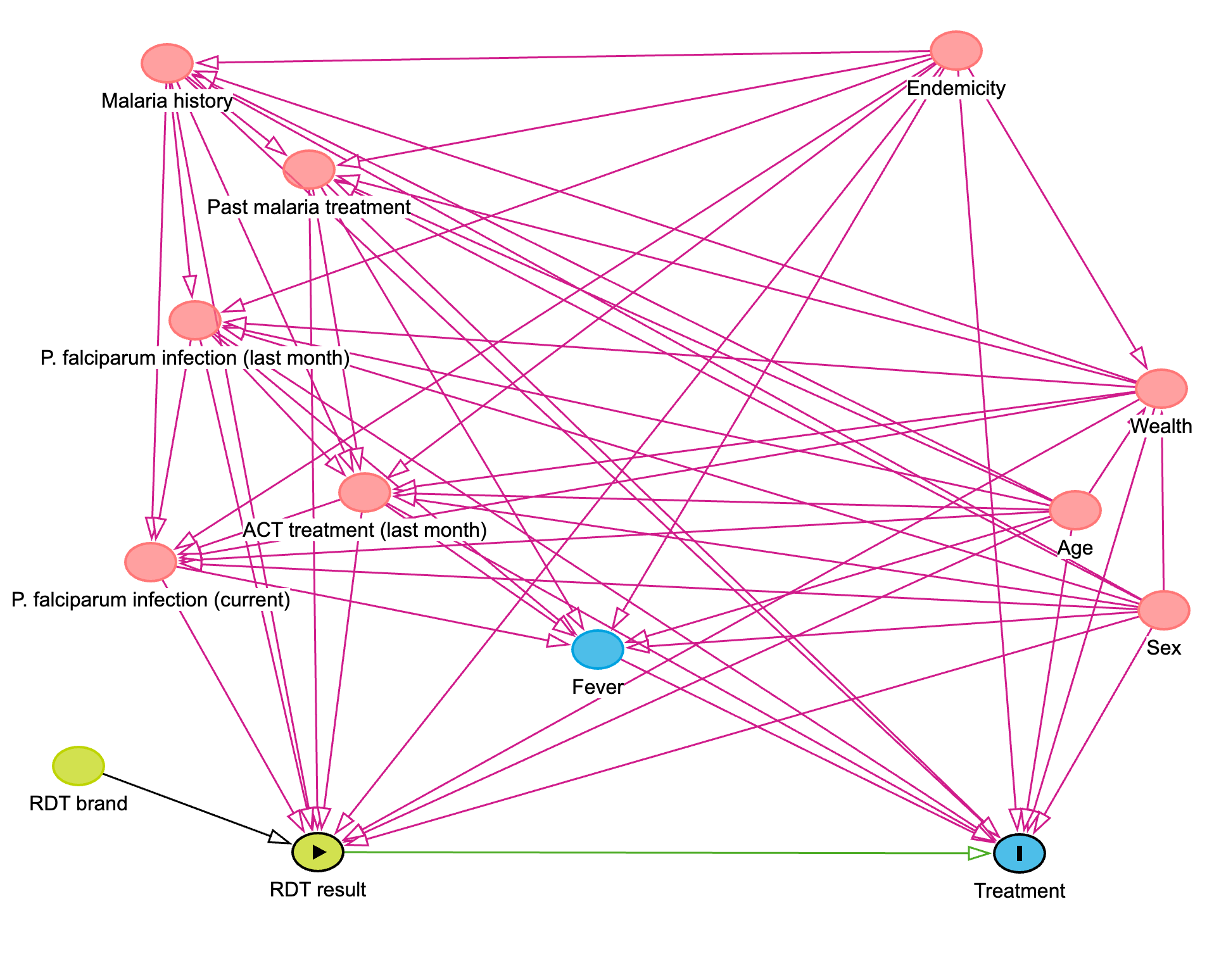


DAGitty was used to create the DAG and identify the minimal sufficient adjustment sets to estimate the total effect of RDT result on treatment:

1. Sex, age, wealth, endemicity, fever, *P. falciparum* infection (last month), artemisinin-based combination therapy or ACT (last month), malaria history, past malaria treatment
2. Sex, age, wealth, endemicity, *P. falciparum* infection (current), *P. falciparum* infection (last month), artemisinin-based combination therapy or ACT (last month), malaria history, past malaria treatment

Inverse probability treatment weights were created using the first adjustment set.

**Figure S2.** Prevalence of *P. falciparum* (by qPCR and rapid diagnostic test, RDT), false-positive RDT, and false-negative RDT during **A**) household and **B)** clinic visits.


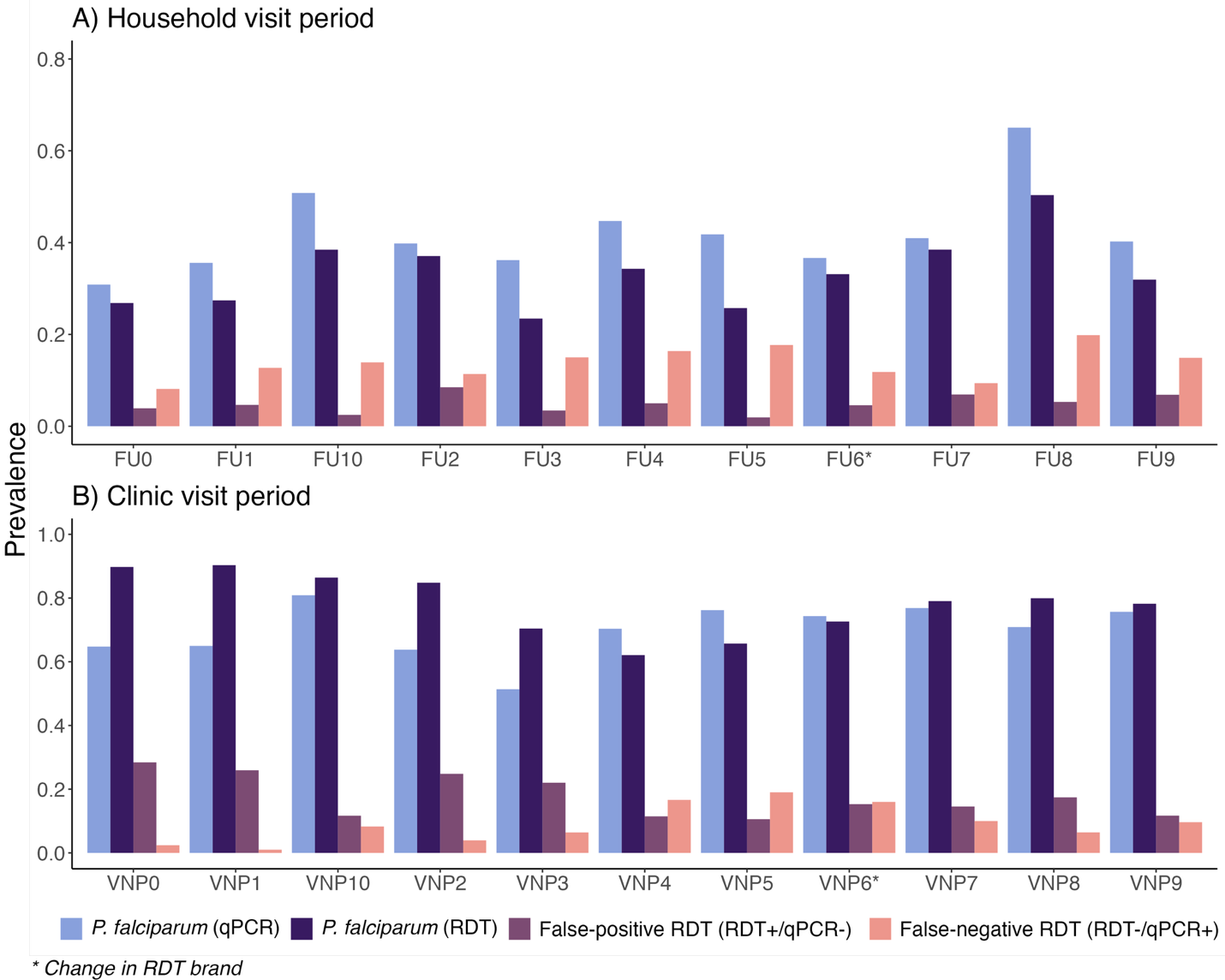


* The switch from SD Bioline RDT detecting falciparum histidine-rich protein 2 (HRP2) and pan-Plasmodium lactate dehydrogenase antigens to CareStart RDT detecting only falciparum HRP2 antigen reflects a change in RDT procurement by the national malaria control program in 2019.

FU = follow-up/household visits (active surveillance); VNP = unscheduled/clinic visits (passive surveillance)

**Figure S3.** Correlates of false-positive and false-negative *P. falciparum* RDT results. False-positive rapid diagnostic tests (RDTs) were defined as RDT-positive but qPCR-negative, and false-negative RDTs were defined as RDT-negative but qPCR-positive – comparing results using complete vs. imputed data [dots = point estimates; horizontal bar around each dot = 95% confidence interval; dotted red line = null value]


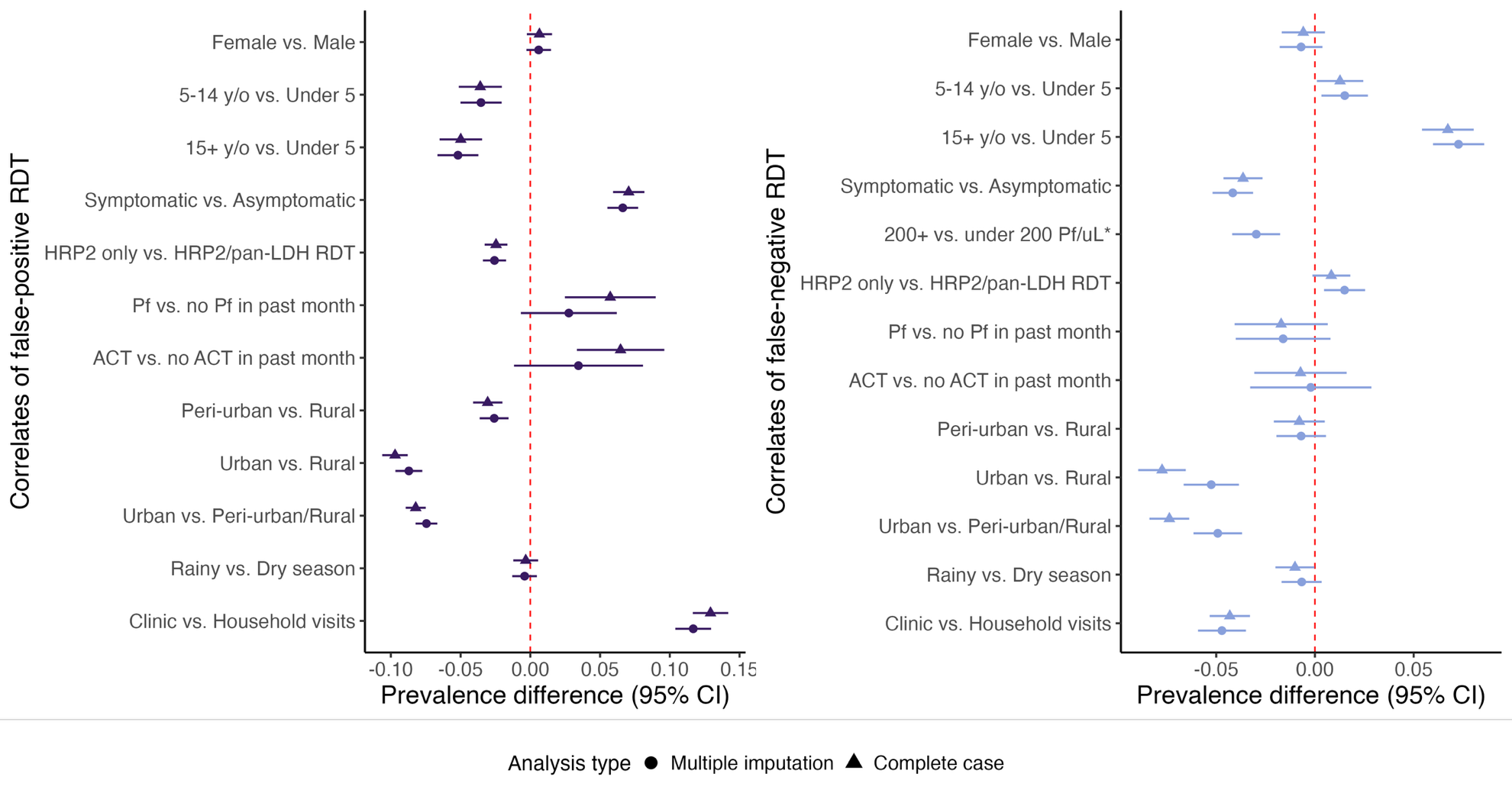


**Figure S4.** Correlates of false-positive (vs. true-negative) and false-negative (vs. true-positive) *P. falciparum* rapid diagnostic tests, reflecting factors associated with diagnostic accuracy

We examined correlates of diagnostic accuracy or performance of malaria rapid diagnostic tests (RDTs) by comparing false-positive and true-negative RDTs to investigate correlates of RDT specificity, and false-negative and true-positive RDTs to investigate correlates of RDT sensitivity. Malaria rapid diagnostic tests (RDTs) were less specific in areas of high transmission intensity, among younger participants and those with a *P. falciparum* infection and treatment in the preceding month when comparing false-positive to true-negative results. RDT sensitivity followed the oppositive pattern; among those with falciparum infections as determined by PCR, older participants in the urban area harboring low parasite-density infections were more likely to have false-negative RDTs.

[dots = point estimates; horizontal bar around each dot = 95% confidence interval; dotted red line = null value]


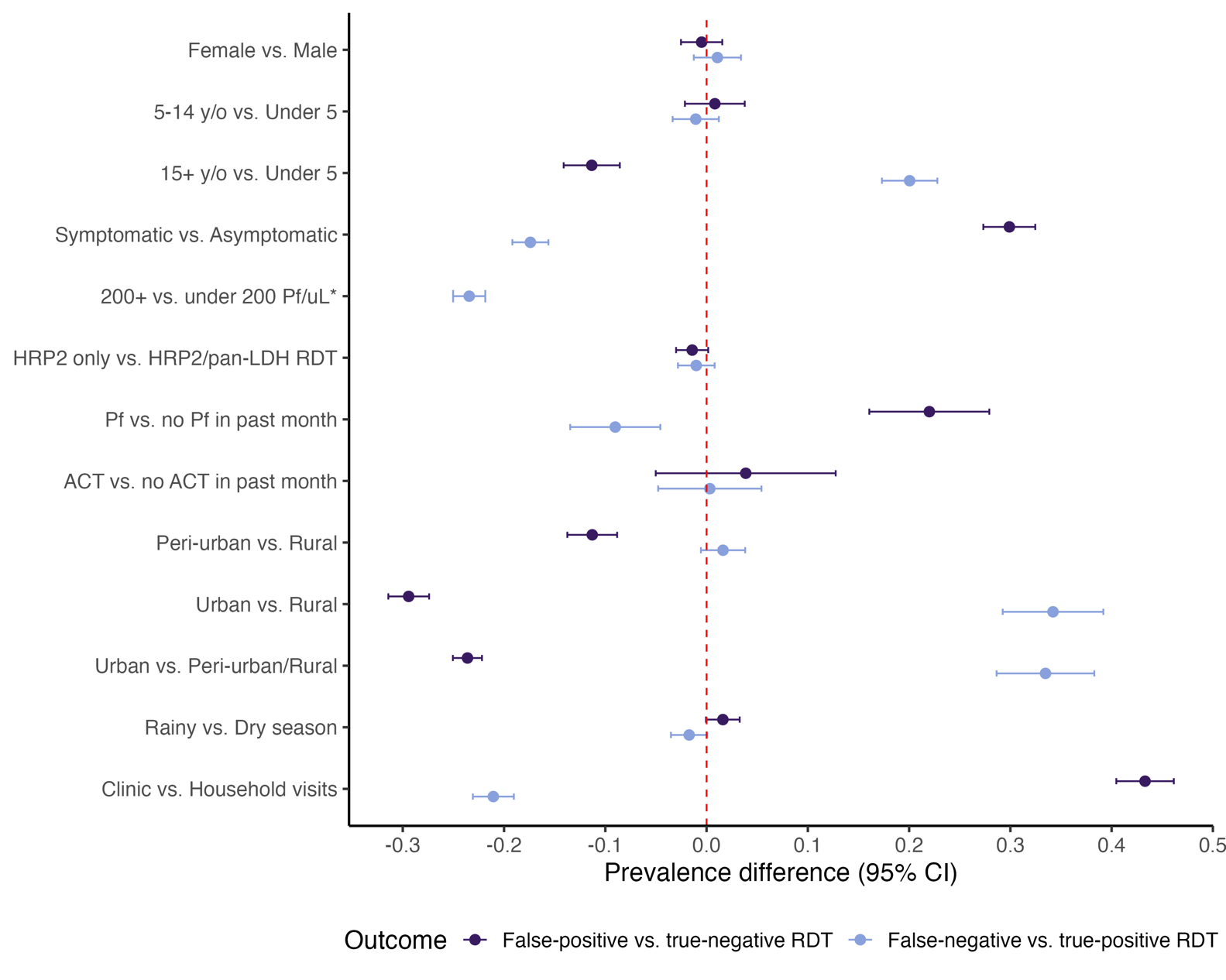


** Parasite densities are absent in the setting of false-positive and true-negative RDT results and therefore cannot be modeled.*

**Figure S5.** Correlates of undertreatment and overtreatment based on RDT results, comparing results from analyses using complete and imputed data.

Overtreatment was defined as treatment administered to rapid diagnostic test (RDT)-negative participants or treatment of participants for whom RDT was not performed. Undertreatment was defined as treatment withheld from RDT-positive participants. Overtreatment was more common during the COVID-19 pandemic and at visits where participants were symptomatic and reported treatment at a prior clinic visit; under-treatment followed the opposite pattern. Both over- and under-treatment were more common in the peri-urban areas.

[dots = point estimates; horizontal bar around each dot = 95% confidence interval; dotted red line = null value]


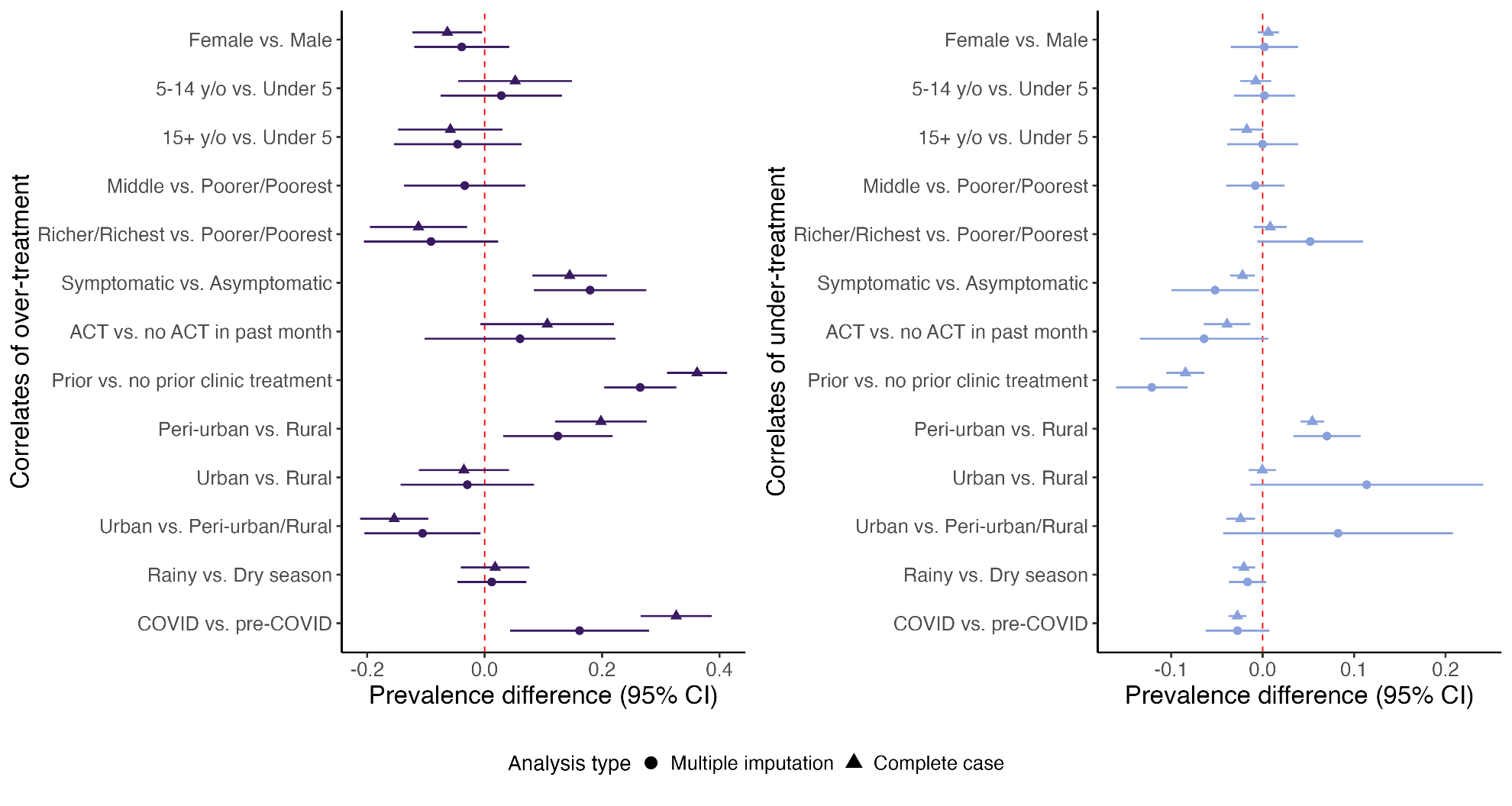


**ALT TEXT**

Figure S1. Directed acyclic graph showing the hypothesized relationship between an exposure, which is rapid diagnostic test result, an outcome, which is treatment, and other factors

Figure S2. Bar plots showing the prevalence of *Plasmodium falciparum* diagnosed by qPCR and rapid diagnostic test, as well as the prevalence of false-positive and false-negative RDT, with prevalence in household visits shown in subfigure A and in clinic visits in subfigure B

Figure S3. Forest plots showing factors associated with false-positive and false-negative RDT results, comparing results obtained using complete vs. imputed data

Figure S4. Forest plots showing factors associated with rapid diagnostic test sensitivity and specificity

Figure S5. Forest plots showing factors associated with over-treatment and under-treatment comparing results obtained using complete vs. imputed data
