# Supplementary figures and images for: Challenges in Malaria Diagnosis and Treatment in Kinshasa Province, Democratic Republic of the Congo
Source: Clin Infect Dis. 2026 Apr 18;83(1):e196–205. doi: 10.1093/cid/ciag262 (PMC13393125; doi:10.1093/cid/ciag262)

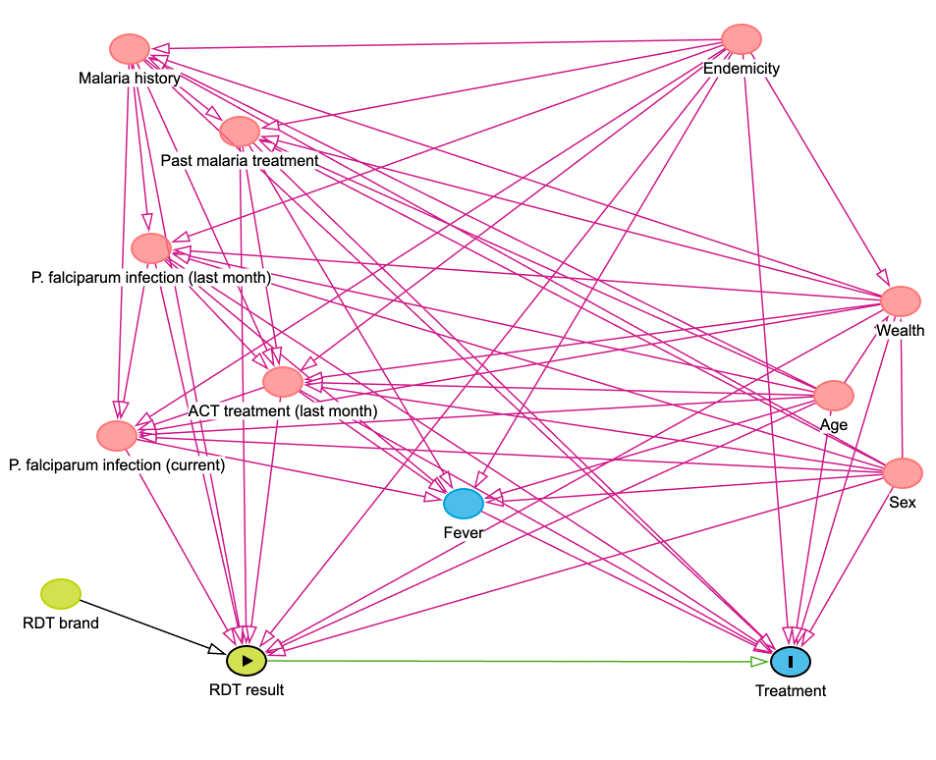

Supplement: ciag262_Supplementary_Data [file ciag262_supplementary_data.zip › Figure S1.tiff]

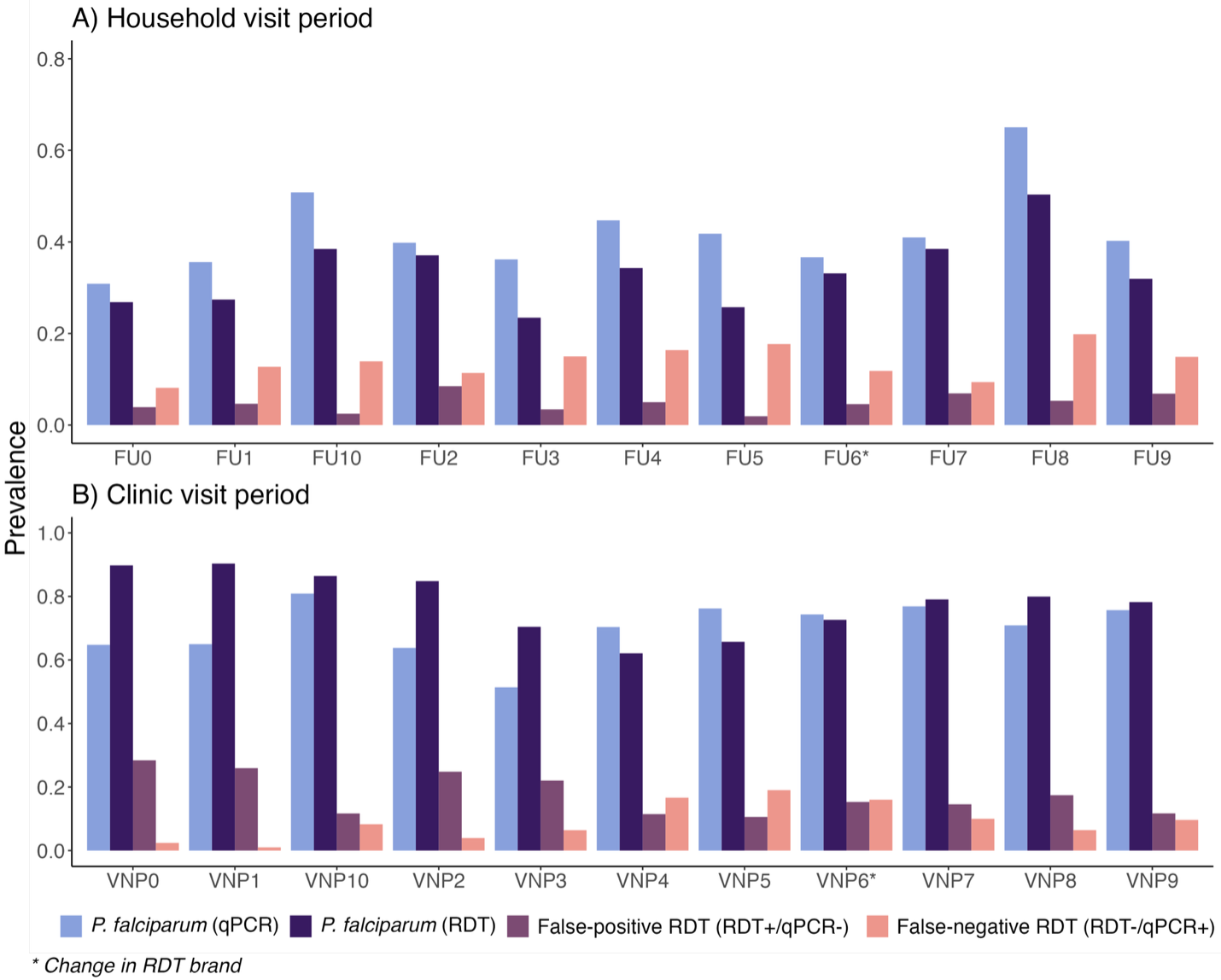

Supplement: ciag262_Supplementary_Data [file ciag262_supplementary_data.zip › Figure S2.tiff]

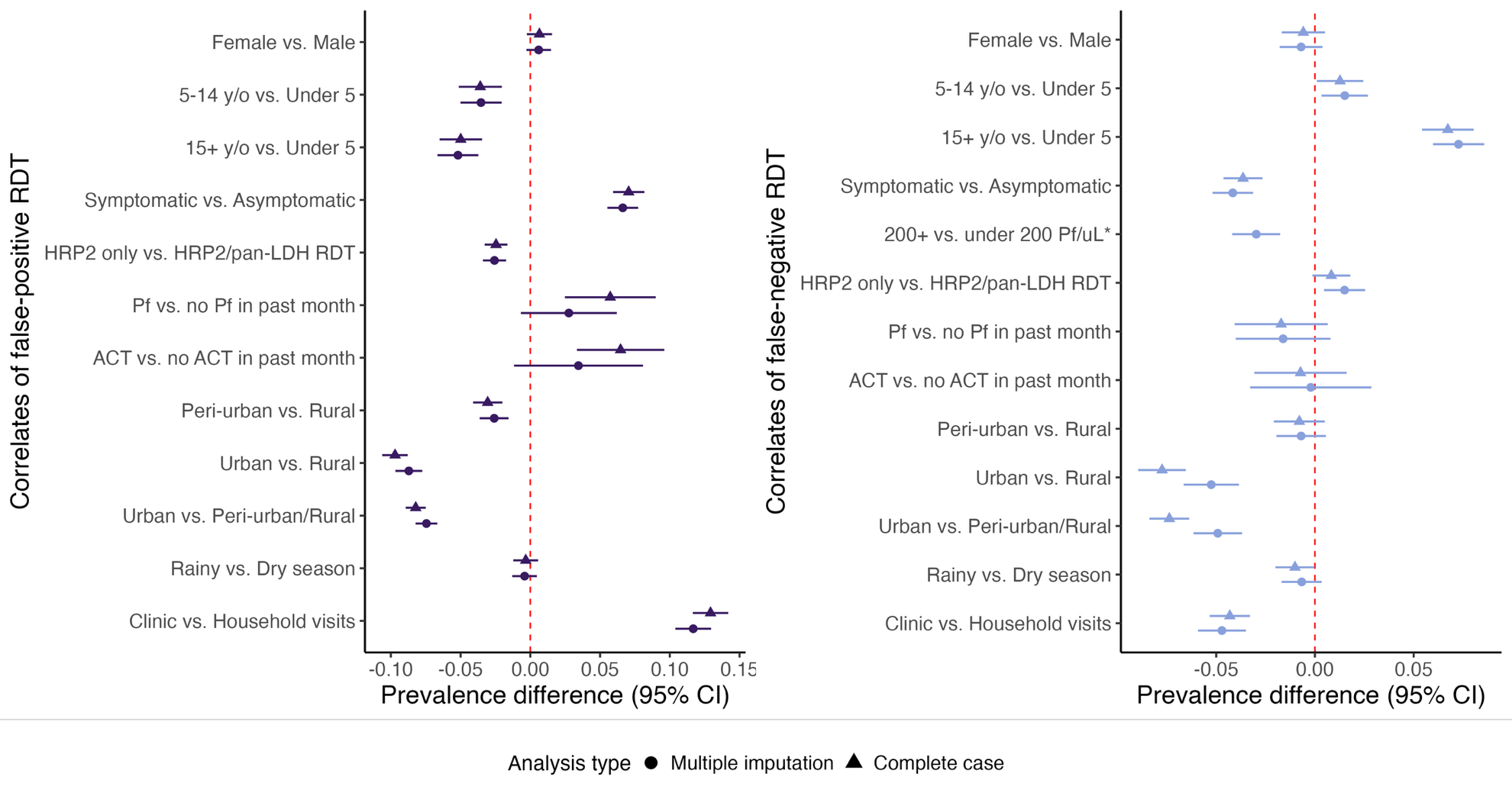

Supplement: ciag262_Supplementary_Data [file ciag262_supplementary_data.zip › Figure S3.tiff]

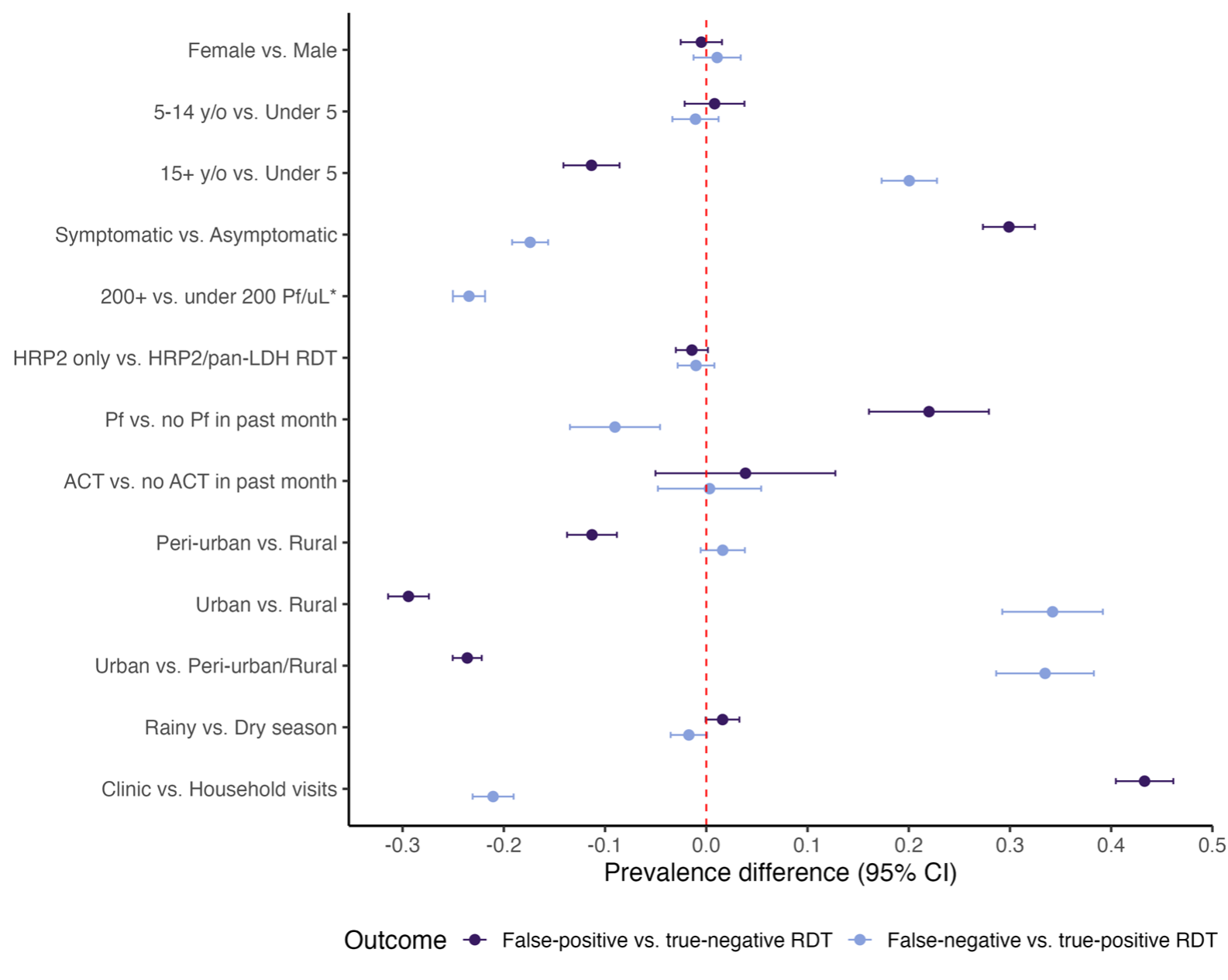

Supplement: ciag262_Supplementary_Data [file ciag262_supplementary_data.zip › Figure S4.tiff]

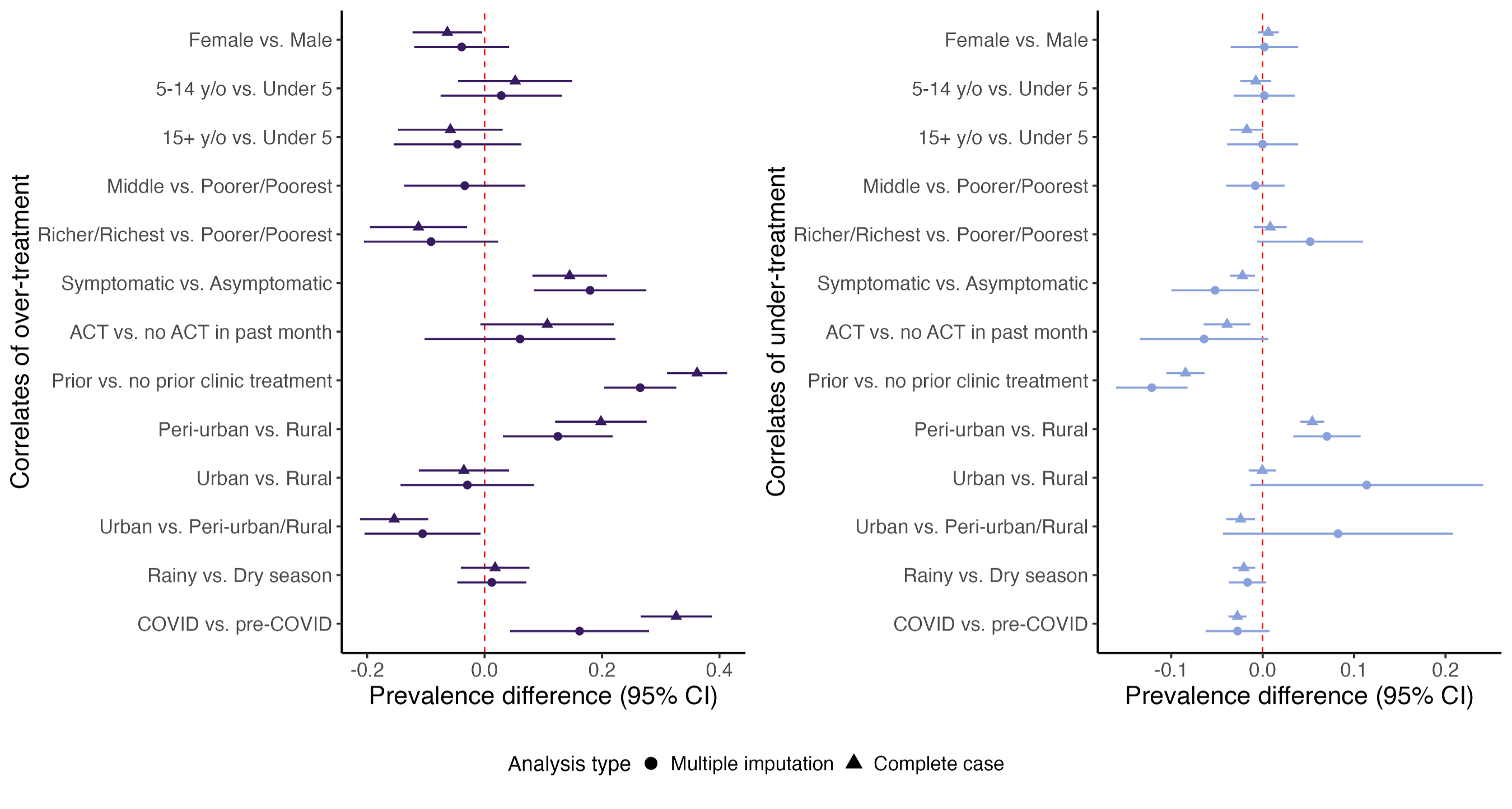

Supplement: ciag262_Supplementary_Data [file ciag262_supplementary_data.zip › Figure S5.tiff]
